# Nasal Obstruction a Factor in the Production of Disease

**Published:** 1917-11

**Authors:** W. Peyre Porcher

**Affiliations:** Charleston, S. C.


### NASAL OBSTRUCTION A FACTOR IN THE PRODUCTION OF DISEASE.

**By W. Peyre Porcher, M.D.,**
**Charleston, S. C.**

It has become a fad of recent years to attribute many diseases of the teeth and tonsils. Like religious fanatics of old who called "fire and sword and desolation a godly thorough reformation," so extremists of today have gone for these organs tooth and nail, or tooth and tonsils, rather and eradicated healthy and unhealthy organs on the bare possibility that pulp stones, rhinoliths, or some other pathological condition might be present. These organs, being visible and accessible, may be made to bear the brunt of ills, imaginary or otherwise. The teeth and tonsils do often become diseased and cause diseases of other organs at a distance. In fact, only recently two striking illustrations have occurred in my practice. The first was a case of rheumatism of the knee joint which had lasted for several months. A culture was taken from one of the crypts of the tonsils from which a pus discharge was exuding. An autogenous vaccine was made and administered hypodermically; the rheumatic pains promptly disappeared and never returned. The second case was that of suspected tuberculosis in a young man age nineteen years. The patient had an afternoon temperature of about 99 degrees F. which had been running for some time. A diagnosis of tuberculosis was made both by x ray and by vaccine, but an abscessed tooth was also found and treated, with the result that the fever promptly stopped and he has gained flesh and can take unlimited exercise without any rise in temperature. Of course, where diseased teeth and tonsils are so manifest the diagnosis should be easily made, but how often must obscure conditions in the deep seated cavities in the nose be overlooked unless the physician is thoroughly competent to make the diagnosis? In this age of medical enlightenment the failure to examine the nose and throat in many of the most common ailments which afflict humanity would scarcely be excusable. This does not apply, as is usually supposed, to diseases affecting the upper respiratory tract alone, because many affections of remorte organs and reflex neuroses, etc., are directly attributed to nasal obstruction and diseases of the lateral sinuses. It has been common in recent years to attribute every case of migraine to some error of refraction. Of course, it is manifestly true that impure air or the improper performance of the function of respirtion, is far oftener productive of headache. As it has been said that headache is the cry of the brain for fresh blood, it must necessarily be true that it is the cry for more purified air.

I have been surprised to find that both graduates and postgraduates often completely ignore the organs of respiration. In fact, it is not uncommon for practioners of medicine to bost of their ignorance of these important organs. There is only one way in which the gravity and importance of diseased conditions in the nose and throat can be recognized and that is by actual experience on the live subject clinically and on the cadaver. Unfortunately written articles and lithographic plates give but a poor impression of the relation of these parts or their clinical significance. Unfortunately also the diseased condition, if situated far back in the nose or in either of the lateral sinuses, cannot be easily demonstrated by one person to another. In fact even although complete relief may be given to a patient by a surgical procedure, it is often very difficult for the surgeon himself to see just what he has done. For example, in order to reach either the thmoid or spenenoid sinuses, the anterior end or in some cases the whole middle turbinate has to be removed. This operation is commonly done, but careful examination is often required to see the parts after the removal of the bone. It would be impossible for me to give an idea of the wonderful results occurring after the restoration of the normal calibre of the nose except by giving the actual testimony of the patients themselves.

Most of these cases are tuberculous pulmonary or bronchial inflammations in which the disease in the nose has either been entirely overlooked, or, worse still, was purposely ignored as being in any way an exciting cause of the inflammatory conditions affecting the organs lower down.

For many years past innumerable papers have been written in regard to the early history and signs of tuberculosis of the lungs and bronchi. Where the early history points to a catarrhal condition in the nose as so commonly occurs, is it not rational to look to that organ for some predisposing cause? Of course, according to the modern teaching of the day. almost every inflammation of the lungs and bronchi is at once attributed either to a tuberculous diathesis or a predisposition to tuberculosis. This word predisposition means only failure or inability to diagnose the exact inciting cause of the disease. We generally say the patient caught a severe cold and acquired tuberculosis, yet how seldom do we make a critical examination of the nose to discover if there was any abnormal condition in the nose which not only rendered the patient more susceptible to what we call a sever cold, but keep up the inflammation so as ot render the patient a fit subject for consumption? No scientific explanation has ever been given of the exact meaning of the words "inherited predisposition." Is it not rational to believe that these words mean a condition in the nose and throat which developed before the actual disease of the lung organ? Although extremely tolerant of diseast, perhaps more so than any other organ, the nose finally yields to repeated invasion and chronic processes set in. These processes may either be a simple cartilaginous spur projecting from the septum or a far more serious purulent condition involving the lateral sinuses.

In the opinion of most authorities and of the writer the presence of a cartilaginous spur is more frequently a factor in the production of acute inflammation in the nose than any other condition. By the mere fact of their presence the nose is rendered far more susceptible to inflammation and repetition of this inflammation results in chronic purulent conditions which end in serious involvement of all the air passages lower down. Is it not reasonable, therefore, that rigid examinations should be made for the presence of such inciting factors in the production of acute inflammation in the nose? Of course all this should be done long before there is any breakage down of lung tissue or persisten bronchitis or tubercule bacilli found in the sputum. I have in mind at present three cases which have more or less recently come under my observation in which this condition was markedly demonstrated.

Cas 1.—A lady had been under the care of a specialist in a neighborhood city for several months for the treatment of pulmonary tuberculosis. On examination I found a large septal spur in her right nostril. As is usual in these cases there was no marked obstruction to respiration in this case or any outward sign of its presence. On that account I judge the specialist in whose care she had been deemed it of no moment and therefore did not urge its removal, or, perhaps as occurs in many instances, being a tuberculous case, he was afraid that the wound might take on a tuberculous condition and refuse to heal. I have known eminent specialists who refused to operate on this ground. It has never been my experience, however, that there was any retardation in the healing process. In this instance the operation was done with the electric trephine and was both bloodless and painless under cocaine and adrenalin. The patient at last reports was getting up at 4 a. m. and performing her household duties.

Case II.—A similar growth was removed from the nostril of a woman whose recovery was so complete that she was, at last report, skipping rope to reduce her flesh. When asked what portion of the treatment she derived the most benefit from she at once replied, "The operation on the nose."

Case III.—A young man not under my immediate care, was sent north for pulmonary tuberculosis. He was in a large sanitarium for many months and returned, having been benefitted but far from well. He stated to me that the growth had been seen and recorded, but that no operation had either been performed or advised for its removal. His attacks of asthma and bronchitis had persisited after his return and he was finally operated on by a specialist in a neighboring State for relief of the nasal condition.

The astonishing fact is that this young man should have been so long under the care of eminent men who entirely ignored the presence of the growth. In this case the catarrhal condition in the nose was so manifest that any one sitting near to the patient could have easily detected it. Chronic purulent sinusitis had set up which needed repeated operations for its correction. The patient was in imminent fear of death before he got in the hands of a surgeon who advised immediate operation. Of course neither the patient or his physician could give credence to the fact that men of such eminence as those in charge of the sanitarium could fail to appreciate the gravity of a purulent ethmoiditis as well as involvement of the surrounding sinuses. It would be needless to allude to atrophic degeneration or scabe formation in the nose where the disease has progressed so far that the function of the nose as a purifier and moistener of the respiration has been almost entirely abolished. Here of course there would be asthma, bronchitis, cough, and finally breaking down of lung tissue. Chills, coryza, wet feet, drafts, etc., are the traditional causes of acute coryza, yet but few physicians look for the more frequent underlying or predisposing causes, namely, pahtlogical condition in the shape of sharp spurs, etc., in the nose itself.

To the bacteriologist, these influences resulting in a hypersecretion of mucus, merely supply a culture medium upon which the bacteria commonly present are able to grow rapidly. Under ordinary circumstances the nasal secretion is considered to be antigermicidal, but an inflammatory secretion, strange to say, is considered to be most excellent medium for the growth of the bacteria and thence increased virulence is not improbable. It is stated that the symptoms of infection from different bacteria vary; certain bacteria, notably Micrococcus catarrhalis and Bacillus foetidus affect chiefly the nose and pharynx, while others find their most convenient lodging place in the lower respiratory organs. Admitting, therefore, the truth of all this, it becomes all the more imperative to investigate all conditions of the inner nostril in any disease affecting the respiratory organs.—New York Medical Journal.

